# The psycho-social effects of COVID-19 on Italian adolescents’ attitudes and behaviors

**DOI:** 10.1186/s13052-020-00833-4

**Published:** 2020-05-24

**Authors:** Carlo Buzzi, Maurizio Tucci, Riccardo Ciprandi, Ilaria Brambilla, Silvia Caimmi, Giorgio Ciprandi, Gian Luigi Marseglia

**Affiliations:** 1 Sociology Department, University of Trento, Trento, Italy; 2 IARD Institute, Milan, Italy; 3 Adolescence Lab Association, Pavia, Italy; 4 Cystic Fibrosis Unit, Istituto G. Gaslini, Genoa, Italy; 5 Department of Paediatrics, Fondazione IRCCS Policlinico San Matteo, University of Pavia, Pavia, Italy; 6 Allergy Clinic, Casa di Cura Villa Montallegro, Via P. Boselli 5, 16146 Genoa, Italy

**Keywords:** COVID-19, Adolescents, Survey, Psycho-social issues, Italy

## Abstract

**Background:**

COVID-19 is an emerging issue that has significant consequences on psycho-social well-being.

**Methods:**

In this regard, a survey was conducted on a large group of adolescents in Italy. The survey investigated four items: concerns and fears, information on the pandemic, provisions of public authorities (e.g., lockdown), and impact on everyday life.

**Results:**

Adolescents actively participated in the survey. COVID-19 affected emotions and lifestyle. COVID-19 influenced relationships with peers and parents. There were regional differences.

**Conclusions:**

The current research highlighted the remarkable, healthy, and certainly unexpected, emotional balance of the new generations in the face of a sudden, unpredictable phenomenon capable of jeopardizing life itself. While understanding the gravity of the phenomenon and willingly adapting to all the necessary precautions, the adolescents still seemed to express an excellent ability to manage situations of insecurity and to deal with unfavorable and adverse conditions by adapting to the new routine and finding alternative and innovative means of meeting their social and psychological needs.

## Background

COVID-19 (coronavirus disease) is greatly influencing our society. Thus it is useful to understand how individuals are experiencing this period of considerable uncertainty.

The pandemic phenomenon, overwhelmingly entering in everyday life, has upset, in a few weeks, the normal behavior of individuals and families. An emergency, such as the COVID-19 pandemic, is an unprecedented experience for everyone, it is not easy to imagine how the adolescents can react. In particular, for today’s teenagers, who live in a generally overprotective social environment, the reaction to the Coronavirus emergency is an unknown factor to which we could respond only *ex-post* [1, 2].

A real-time survey was conducted to try to respond to this question on a large group of adolescents across Italy. The aim was to detect the psycho-social effects induced by the spread of COVID-19 and the provisions put in place by national and local public authorities in the particular target of adolescents.

## Methods

This cross-sectional study was performed during March 2020. It involved a nationally representative sample of 2064 adolescents. Data were collected from the survey titled “Teenagers and COVID-19” as part of the Survey On Lifestyles of Teenagers conducted by the “Italian Society of Adolescent Medicine” and the “Adolescence Laboratory” Association, in collaboration with the Pediatric Department of the University of Pavia.

Random selection of school classes was performed based on a national stratified multistage (data from the Italian Ministry of Education University and Research) for secondary schools, first degree), according to a factorial design that considered the geographic distribution (North-East; North-West, Central Italy, South, Islands) and, within each area, the size of the population.

Students were invited to fill in all the sections of the questionnaire, which were applicable. According to the Italian laws and good ethical practice, a written and informed consent of the parents was required before participation in the study. The Ethics Committee of the Medical Faculty of the University of Pavia accepted the study protocol.

Four thematic areas were investigated through a short online questionnaire: their ​​concerns and fears, the area of information, the area of the provisions to contrast, the area of repercussions on everyday life. The analysis investigated the usual socio-personal variables such as age, gender, and geographical location. We considered a time index (aimed at understanding how the assessments changed during the detection in conjunction with the increase in the spread of the virus). We also included an index of severity (reuniting the regions of residence about the level of the contagion rate).

## Results

The results provided interesting information about the adolescents’ reactions to COVID-19.

As regards concerns and fears, the majority of adolescents declared to be: worried but not too much, a third “little” or “not at all” (Table 1). In this sense, the adolescents’ tendency, more in males than females, to a certain underestimation of the danger was confirmed. In this respect, they reported that they are less anxious than their parents (Table 2). The adolescents, as are approaching adulthood, the apprehension increased, and young people were more realistic by sharing more of the same fears as family adults (Table 3) [3]. Two interesting trends appeared. The first is that the level of concern, recorded after the first four days of detection, increased significantly (from the fourth to the eighth day), but then it remained constant (Table 4). After an initial understandable increase (which coincided largely with the entry into force of the restrictions throughout the country), the new situation was metabolized, returning to a sort of “normality.” The second interesting result was that the concern expressed by adolescents (residing in the central-southern regions, and less involved in the diffusion of the phenomenon) was double that of their peers from the northern regions (Table 5). This outcome probably depended on the fact that in the north, the emergency was already being experienced in full. However, in the rest of the country, much less affected by the epidemic, the unknown for an indeterminate future generated greater fear. In other words, individual and collective reactions to COVID-19 could be explained by the fear of the unknown. The representation of unknown danger, of which there is no awareness but which is seen as probably imminent, triggered a superior emotional reaction compared to those who have the possibility, or even the illusion, to better control an adverse situation for the simple fact to be in direct contact with it. Even if everyone agreed that the emergency would last for months (Table 6) [4].

**Table 1:** Table of contingency of degree of concern

		Gender		Total
		Female	Male	
Degree of concern	nothing	2.6%	8.2%	4.7%
	little	24.1%	38.4%	29.6%
	moderately	54.9%	45.2%	51.2%
	a lot	18.3%	8.2%	14.5%
Total		100%	100%	100.0%

**Table 2:** Table of contingency of who is more concerned among you and your parents

		Gender		Total
		Female	Male	
Who is more concerned among you and your parents?	definitely me	2.7%	22%	2.5%
	in the same way	48.3%	349%	43,1%.
	definitely my parents	49.1%	629%	54.4%
Total		100%	100%	100%

**Table 3:** Table of contingency of degree of concern

		Age				Total
		Under 13	14–16	17–19	Over 20	
Degree of concern	nothing	7.2%	5.5%	3.8%	2.8%	4.7%
	little	27.8%	33.6%	27.8%	16.4%	29.6%
	moderately	4.,9%	49.0%	55.1%	49.3%	51.2%
	a lot	21.1%	11.9%	13.3%	3.,5%	14.5%
Total		100%	100%	100%	100%	100%

**Table 4:** Table of contingency of degree of concern in the interview period

		Interview period			Total
		9th–12th March	13th -16th March	17th -20th March	
Degree of concern	nothing	4.9%	3.3%	5.1%	4.7%
	little	31.3%	25.7%	24.2%	29.6%
	moderately	50.2%	55.1%	53.7%	51.2%
	a lot	13.7%	15.8%	17.0%	14.5%
Total		100%	100%	100%	100%

**Table 5:** Table of contingency of degree of concern in the Central-North vs South-Isles

		Italy		Total
		Central-North	South-Isles	
Degree of concern	nothing	5%	4.3%	4.8%
	little	32.2%	23.4%	29.6%
	moderately	51.7%	50.2%	51.2%
	a lot	11.1%	22.2%	14.4%
Total		100%	100%	100%

**Table 6:** Table of contingency of prediction of emergency duration Central North South. % in central north south

		Italy		Total
		Central-North	South-Isles	
Prediction of emergency duration	days	0.2%	0.2%	0.2%
	weeks	21.7%	23.6%	22.3%
	months	77.0%	74.8%	76.3%
	years	1.2%	1.4%	1.2%
Total		100%	100%	100%

As regards information, during the timing of the survey, the judgment on the information conveyed by the mass media considered by a relative majority of the respondents as “correct and adequate” but also, by two substantial minorities, as “too alarming” (one third) or “reticent for do not frighten” (one fifth) as reported in Table 7. Wide distrust, however, was experienced towards information coming from social networks for its inaccuracy, if not for its total unreliability (Table 8). However, the level of discussion in the family remained very high (four-fifths of young people often talk about it with their parents), the medium with friends (half speaks often) and low that with teachers (less than a quarter). However, while in the case of parents and teachers this level remains constant over time, the discussion with friends decreases considerably, almost halving, passing from the first days of survey (9–12 March) to the last (17–20 March) as saying that the interest of the topic in peer discussions is reduced and left space to other issues (Tables 9, 10, 11). This response was a sign of the great ability to metabolize events, albeit dramatic, shown by adolescents (Table 12).

**Table 7:** Table of contingency of judgement of mass-media information in the interview period

		Interview period			Total
		9th–12th March	13th -16th March	17th -20th March	
Judgement of mass-media information	too much alarming	36.9%	**31.4%**	34.8%	36.0%
	correct and balanced	43.7%	**48.2%**	46.8%	44.6%
	hide reality not to scare	19.4%	**20.5%**	18.4%	19.4%
Total		100%	**100%**	100%	100%

**Table 8:** Table of contingency of judgement of social network information in the interview period

		Interview period			Total
		9th–12th March	13th -16th March	17th -20th March	
Judgement of social network information	trustworthy	17.7%	14.6%	15.3%	17.0%
	inaccurate	72.1%	70.8%	70.6%	71.8%
	mainly fake	10.2%	14.6%	14.1%	11.2%
Total		100%	100%	100%	100%

**Table 9:** Table of contingency of talks about emergency with parents in the interview period

		Interview period			Total
		9th–12th March	13th -16th March	17th -20th March	
Talks about emergency with parents	often	79.7%	82.5%	76.0%	79.4%
	sometimes	17.8%	14.9%	20.5%	17.9%
	rarely	2.1%	2.0%	3.1%	2.2%
	never	0.5%	0.7%	0.4%	0.5%
Total		100%	100%	100%	100%

**Table 10:** Table of contingency of talks about emergency with friends in the interview period

		Interview period			Total
		9th–12th March	13th -16th March	17th -20th March	
Talks about emergency with friends	often	60.2%	58.1%	37.8%	56.4%
	sometimes	30.8%	36.3%	46.2%	33.8%
	rarely	7.4%	4.6%	12.9%	8%
	never	1.6%	1.0%	3.1%	1.8%
Total		100%	100%	100%	100%

**Table 11:** Table of contingency of talks about emergency with teachers in the interview period

		Interview period			Total
		9th–12th March	13th -16th March	17th -20th March	
Talks about emergency with teachers	often	23.7%	25.9%	21.2%	23.5%
	sometimes	41.5%	45.5%	50.9%	43.4%
	rarely	22.6%	17.9%	19.8%	21.7%
	never	12.2%	10.6%	8.1%	11.4%
Total		100%	100%	100%	100%

**Table 12:** Table of contingency of talks about emergency with friends considering Age

		age				Total
		Under 13	14–16	17–19	Over 20	
Talks about emergency with teachers	often	22.5%	50.5%	65.4%	75.0%	56.4%
	sometimes	45.0%	38.2%	29.1%	22.6%	33.8%
	rarely	24.8%	9.4%	4.7%	1.4%	8%
	never	7.7%	1.9%	0.8%	0.9%	1.8%
Total		100%	100%	100%	100%	100%

As concerns the provisions of public authorities, such as the lockdown, the consensus for disease prevention and contrast provisions was very high and increased, as far as possible, even over time, becoming almost unanimous (exceeding 94%), as reported in Table 13. Similarly, after an initial uncertainty in the first days of the survey, an overwhelming majority (79%) said that they were always “respected” the provisions and only a minority, equal to one fifth, said they respected them but with some transgressive derogations (Table 14).

**Table 13:** Table of contingency of judgement about precautions indicated by authorities in the interview period

		Interview period			Total
		9th–12th March	13th -16th March	17th -20th March	
Judgement about precautions Indicated by authorities	exaggerated	3.9%	2.3%	1.8%	3.4%
	correct	86.6%	92.4%	93.2%	88.2%
	I can’t judge	9.5%	5.3%	5.1%	8.4%
Total		100%	100%	100%	100%

**Table 14:** Table of contingency of observance of precautions in the interview period

		Interview period			Total
		9th–12th March	13th -16th March	17th -20th March	
Do you observe precautions?	Yes, ever	48.9%	78.5%	78.9%	56.5%
	sometimes	47.9%	21.5%	20.7%	41.0%
	rarely	2.7%		0.4%	2.0%
	never	0.6%			0.4%
Total		100%	100%	100%	100%

As regards everyday life, most adolescents were “Resilient,” such as rebuild their habits and social network without major dramas, even while staying at home. It was what emerged from the last area investigated, such as concerning the repercussions on everyday life and relationships caused by the provisions of the authorities and the consequent restriction of individual freedom. Of course, great help came both from the school that organized itself to continue the lessons online and from the social platforms. The importance of having continued online schooling went beyond what can be didactically effective, but it was linked to the social function that school plays for adolescents [5]. Furthermore, being able, through this device, to reconstruct a sort of daily “agenda” was certainly helping the overall balance of the children. “It is not all the same as before,” said the teenagers, “we meet less,” but “we communicate more through social networks.” Fewer written or vocal messages, but many video calls, to finally recover the pleasure of dialogue and, above all, to continue seeing each other (Table 15). The males seemed to suffer most from the situation. Over a quarter of the male champion complained that he had contracted his social relationships with friends, while less than a fifth of the girls say the same thing. The obvious consequence for the fact that, at that age, male sociality had always developed more “outdoor” than females (Table 16).

**Table 15:** Table of contingency of thinking if there will be negative consequences in your school education considering gender

		Gender		Total
		Female	Male	
Do you think there will be negative consequences in your school education?	a lot	40.7%	30.5%	36.8%
	a few	30.4%	34.0%	31.8%
	no-one	2.9%	7.0%	4.5%
	I don’t know	26.0%	28.6%	27.0%
Total		100%	100%	100%

**Table 16:** Table of contingency of changing in friend relationship considering the age

		age				Total
		Under 13	14–16	17–19	Over 20	
Changing in friend relationship	no	12.6%	8.9%	6.7%	8.5%	8.2%
	less communication and less meeting	27.0%	20.2%	23.0%	25.1%	22.2%
	more communication via social network but less meeting	60.4%	70.8%	70.3%	66.4%	69.6%
Total		100%	100%	100%	100%	100%

Concerning school - although the activity has somehow resumed “remotely” - there is widespread concern about how much this emergency can have negative effects on their school preparation. Especially among girls, traditionally more attentive to school performance (Table 17) and among students who have to face the final exam (Table 18) [6].

**Table 17:** Table of contingency of changing in friend relationship considering the gender

		Gender		Total
		Female	Male	
Changing in relationship with friends	no	6.5%	11.0%	8,.%
	less communication and less meeting	19.4%	26.7%	22.2%
	more communication via social network but less meeting	74.1%	62.3%	69.6%
Total		100%	100%	100%

**Table 18:** Table of contingency of thinking if there will be negative consequences in your school education considering the age

		age				Total
		Under 13	14–16	17–19	Over 20	
Do you think there will be negative consequences in your school education?	a lot	32.4%	33.9%	41.8%	28.6%	36.8%
	a few	33.8%	33.3%	30.3%	28.1%	31.8%
	no-one	5.0%	4.4%	3.8%	9.0%	4.5%
	I don’t know	28.8%	28.4%	24.1%	34.3%	27.0%
Total		100%	100%	100%	100%	100%

## Discussion

The current research highlighted the remarkable and undoubtedly unexpected, emotional balance of the new generations in the face of a sudden, unpredictable phenomenon capable of jeopardizing life itself. COVID-19 pandemic is significantly affecting the emotional and behavioral experience in Italian adolescents. Adequate information and counseling could give helpful support to cope with this issue.

While understanding the gravity of the phenomenon and willingly adapting to all the necessary precautions, the adolescents still seemed to express an excellent ability to live situations of insecurity and to deal with unfavorable and adverse conditions by seeking conditions of new normalcy and finding alternative solutions of daily life.

We could probably explain this phenomenon as this new generation, much more than the previous ones, was subjected to the stress of existential precariousness and uncertainty of the future. It led them, much more than adults, to adapt to the incessant and rapid rhythms with which our daily lives are transformed. We could, therefore, hypothesize that thanks to this sort of “vaccine,” adolescents and young adults were proving somewhat more adequate than their “fathers” to respond to this new event that suddenly appeared in the life of all of us.

On the other hand, the current report has some limitations, including the absence of comparing literature, the lack of psychometric questionnaires or scales, the statistics were only descriptive, and the survey was cross-sectional.

## Conclusions

COVID-19 significantly affects the emotional and lifestyle of Italian adolescents. Therefore, the adequate initiative should be adopted to inform and support adolescents in this period.

## Abbreviation

COVID-19: Coronavirus disease 2019

## References

[1] Rankin K, Sweeny K, Xu S (2019). Associations between subjective time perception and well-being during stressful waiting periods. Stress Health.

[2] Pawluk EJ, Koerner N (2016). The relationship between negative urgency and generalized anxiety disorder symptoms: the role of intolerance of negative emotions and intolerance of uncertainty. Anxiety Stress Coping.

[3] Main A, Lougheed JP, Disla J, Kashi S (2019). Timing of adolescent emotional disclosures: the role of maternal emotions and adolescent age. Emotion.

[4] Wang Wenchao, Wu Xinchun, Lan Xiaoyu (2020). Rumination mediates the relationships of fear and guilt to posttraumatic stress disorder and posttraumatic growth among adolescents after the Ya’an earthquake. European Journal of Psychotraumatology.

[5] Guo J, Liu L, Zhao B, Wang D (2020). Teacher support and mental well-being in Chinese adolescents: the mediating role of negative emotions and resilience. Front Psychol.

[6] Kim KM (2020). What makes adolescents psychologically distressed?.

